# Genomics and High-Resolution Typing Confirm Predominant Clonal Evolution down to a Microevolutionary Scale in *Trypanosoma cruzi*

**DOI:** 10.3390/pathogens9050356

**Published:** 2020-05-08

**Authors:** Michel Tibayrenc, Francisco J. Ayala

**Affiliations:** 1 Maladies Infectieuses et Vecteurs Ecologie, Génétique, Evolution et Contrôle, MIVEGEC (IRD 224-CNRS 5290-UM1-UM2), Institut de Recherche pour le Développement, BP 34394 Montpellier CEDEX 5, France; 2 Catedra Francisco Jose Ayala of Science, Technology, and Religion, University of Comillas, 28015 Madrid, Spain; fjayala2018@gmail.com

**Keywords:** Chagas disease, parasitic protozoa, clonality threshold, genetic recombination, phylogenetic signal, Russian doll pattern

## Abstract

*Trypanosoma cruzi*, the agent of Chagas disease, is a paradigmatic case of the predominant clonal evolution (PCE) model, which states that the impact of genetic recombination in pathogens’ natural populations is not sufficient to suppress a persistent phylogenetic signal at all evolutionary scales. In spite of indications for occasional recombination and meiosis, recent genomics and high-resolution typing data in *T. cruzi* reject the counterproposal that PCE does not operate at lower evolutionary scales, within the evolutionary units (=near-clades) that subdivide the species. Evolutionary patterns in the agent of Chagas disease at micro- and macroevolutionary scales are strikingly similar (“Russian doll pattern”), suggesting gradual, rather than saltatory evolution.

## 1. Preliminary Recalls about the Predominant Clonal Evolution (PCE) Model

The predominant clonal evolution (PCE) pattern does not mean that genetic recombination is either absent, or of little evolutionary significance [1], but rather, that it is not effective enough to erase a persistent and highly detectable phylogenetic signal at all evolutionary scales. The definition of clonality in PCE is therefore based on severe restriction to genetic recombination, a definition that is shared by many authors working on pathogen population genetics (see many references in [2]). The criteria selected for stating that the phylogenetic signal is reliable are the classic, widely accepted, means used in the articles analyzed by us in the present study—(i) mutual corroboration by different markers (see Table 1 in [3]); (ii) posterior probabilities when Bayesian analysis is concerned; (iii) bootstrap, with the limit value of 0.70 considered as significant [4].

PCE is therefore not rejected by the sole detection of genetic exchange, hybridization and meiosis [5,6]. As recalled many times [7], the PCE model is compatible with such traits. Which makes it possible to definitely and specifically challenge the PCE hypothesis is the absence of a stable phylogenetic signal at any evolutionary scale and a population structure that meets panmictic expectations, particularly lack of a statistically significant linkage disequilibrium (nonrandom association of genotypes occurring at different loci) [7]. 

We have coined the term “near-clades” [8] to designate, within pathogen species, genetic subdivisions that are discrete and stable, but that could be somewhat clouded by occasional genetic exchange. As a matter of fact, “true” clades are supposed to be strictly separated from each other. Now in virtually all pathogen species, even if PCE obtains, as noted above, occasional bouts of genetic exchange are recorded. The term “clade” therefore is not adequate. 

## 2. *Trypanosoma cruzi* and the PCE Model

*Trypanosoma cruzi* is the parasite responsible for Chagas disease in the New World. It has been the object of early, pioneering studies dealing with its isoenzyme variability, making it possible to characterize its strains [9]. The interpretation of this isoenzyme diversity in population genetic terms has made it possible to propose that this parasite has a predominantly clonal population structure [10]. The evidence for it is as follows—at the level of the whole species, several multilocus genotypes occur at frequencies that are at variance with panmictic expectations, and are widely distributed in various ecosystems and hosts. A highly significant linkage disequilibrium is recorded [10]. The species is subdivided into at least six main “discrete typing units” or DTUs [11,12], namely Tc I to VI. Evolutionary speaking, these DTUs amount to near-clades [8]. More recently, an additional discrete typing unit/near clade has been described under the name of TcBat. It has been isolated exclusively from bats and is widespread over vast geographical areas and time spans [12]. The available data do not make it possible to test our PCE model within TcBat.

## 3. *T. cruzi* PCE Challengers

**Figure 1:**
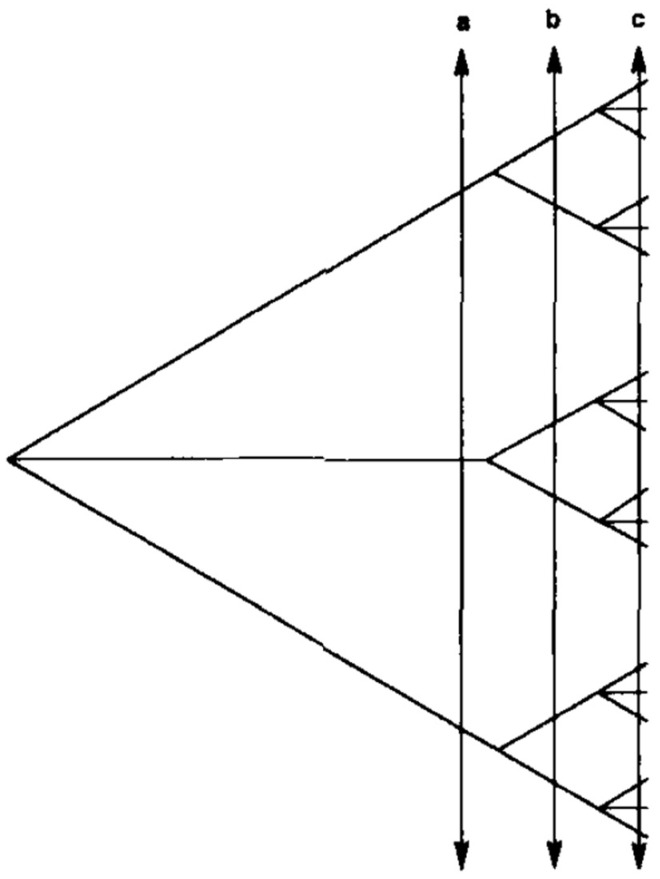
The impact of marker resolution on population genetics and phylogenetic analysis. If a marker with low resolution is used (a), the lesser genetic subdivisions of the species (right part of the figure) will show limited or null genetic variability, which may make it impossible to reject the null hypothesis of panmixia, due to a statistical type II error (after [16]).

**Figure 2:**
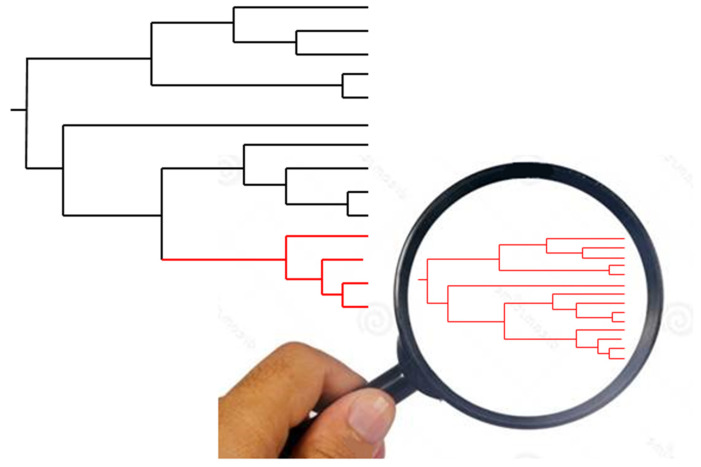
“Russian doll” model. When population genetic analysis is performed with adequate markers (of sufficient resolution) within each of the near-clades that subdivide the species under study (large tree, left), they reveal a miniature picture of the whole species, with the two main predominant clonal evolution features, namely, linkage disequilibrium and lesser near-clades (small tree, right). This is evidence that within the near-clades, predominant clonal evolution also operates (after [2]).

Obstacles to genetic recombination and the presence of a ubiquitous, stable, phylogenetic signal at the level of the whole *T. cruzi* species is no longer under debate. However, the PCE model in *T. cruzi* has been challenged with two lines of arguments, namely—(i) it is based on outdated markers that lack resolution [13]. This is not a valid argument—markers that lack resolution should favor the null hypothesis of panmixia (random genetic exchange) through a mechanism of statistical type II error (impossibility to reject the null hypothesis, not because this null hypothesis is true, but because of a lack of resolution of the used means to test it) rather than the working hypothesis of clonality (Figure 1). (ii) The presence of genetic subdivisions (=“near-clades”) within *T. cruzi* would be “self-evident”, which amounts to saying that the outcome of any population genetics and phylogenetic analysis is self-evident. Evidencing obstacles to recombination at the level of the whole species is therefore trivial and vain [14]. However, high-resolution genomic typing will show that similar patterns of obstacles to genetic exchange are not recorded at lower evolutionary scales, under the level of the near-clades [14]. This last argument aims at specifically challenging the “Russian doll model” [15], which states that PCE is verified at all evolutionary scales, and within-near-clade population structure is a miniature form of the population structure of the whole species (Figure 2).

**Figure 3:**
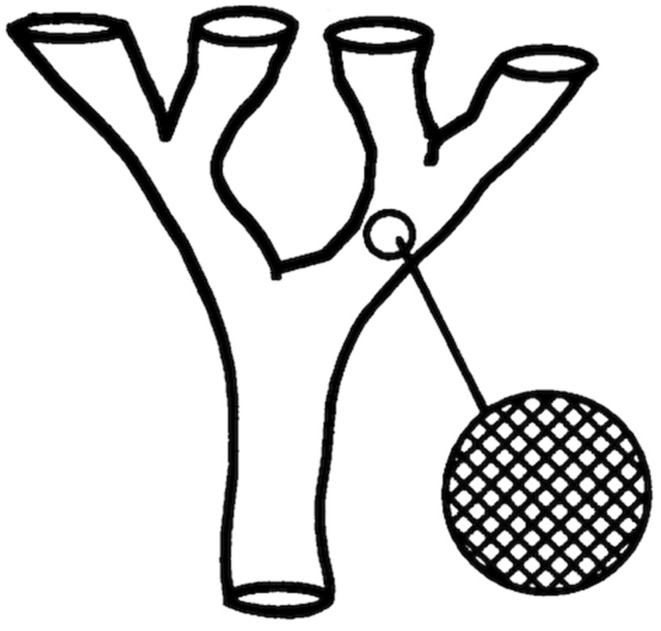
Cryptic biological speciation: the evolutionary lines that subdivide the species are genetically isolated from each other. However, within each of them, genetic recombination occurs randomly, except when physical obstacles (space and/or time) occur (after [18]).

**Figure 4:**
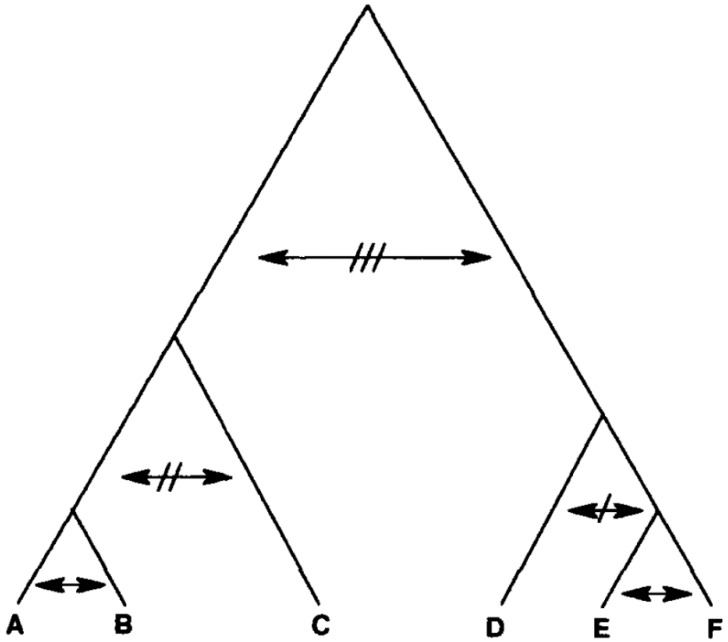
“Progressive clonality”. The frequency of genetic exchange is inversely proportional to the evolutionary distance between any two different genotypes. It is virtually random among identical or very closely related genotypes (homogamy, selfing) and is progressively inhibited as genetic distances increase (after [16]).

At this microevolutionary level, within each of the main genetic clusters (near-clades) that subdivide the species, two evolutionary models would imply that the Russian doll pattern is not verified. They both deal with lack of restriction to genetic recombination:

(a) Biological speciation—each of the near-clades correspond to cryptic species that are genetically isolated from each other, but within which genetic exchange is random, except for physical obstacles (time and/or space) to this random gene flow (see Figure 3). This hypothesis of speciation has been invoked to claim that the main subdivisions (Savannah, Killifi, Forest) within *Trypanosoma congolense* are not evidence for PCE, because they could correspond to cryptic “species”. However, the authors did not clearly refer to a model of biological speciation [17]; and

(b) Progressive clonality—this situation refers to the case where the amount of genetic exchange is inversely proportional to the evolutionary distance between any two given genotypes [16]. If the genotypes are either identical or very similar, genetic exchange is abundant (homogamy, selfing). If they are distantly related, genetic exchange is either severely limited or lacking (Figure 4). Such an evolutionary model is believed to be frequent in bacteria [19].

It is clear that, first, (a) and (b) mean that genetic recombination is not limited or is poorly limited at microevolutionary scales (under the level of the near-clade); second, the means to distinguish the Russian doll model from either (a) or (b) is to give evidence for the presence of PCE traits (linkage disequilibrium and, most of all, constant phylogenetic signal—see Figure 2) within each of the near-clades that subdivide the species under study. However, this demands the use of genetic markers with a sufficient resolution. If this is not the case, lack of resolution of the markers could lead to a wrong hypothesis of panmixia due to a statistical type II error (see Figure 1).

## 4. New Analyses with High-Resolution Typing Challenge the Challengers

Our previous articles did already include the analysis of studies based on high-resolution markers and genomics data. However, to address the criticisms that (i) our model is based on outdated markers that lack resolution [13]; (ii) our model will not be verified at lower evolutionary scales [14], we have reconsidered the problem of PCE in *T. cruzi* in the light of numerous new published articles. This makes it possible to reliably test the Russian doll model within *T. cruzi* near-clades, and to illustrate some important aspects of the PCE model that are frequently misunderstood. 

A wealth of studies show that within the near-clade TcI, in various countries, Russian doll patterns with a highly detectable phylogenetic signal are present. This is against the hypotheses of biological speciation (Figure 3) and progressive clonality (Figure 4).

**Figure 5:**
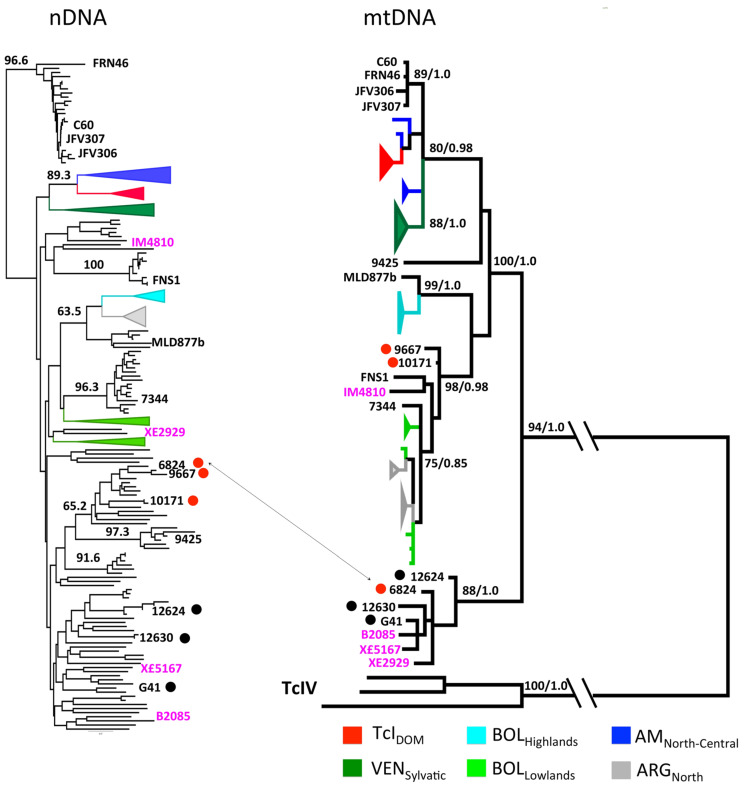
Double phylogenetic tree based on nuclear genes (**left**) and mitochondrial genes (**right**) in a sample of TcI Brazilian strains (after [20]). The TcI discrete typing unit/near clade, itself a discrete subdivision of the species *T. cruzi*, is clustered into various lesser near-clades. Several of these lesser near-clades are supported by significant bootstrap values (numbers along the branches); example—top lesser near-clade—bootstrap 96,6.

In the Atlantic forest region of Brazil, the analysis of 107 wild strains, all identified as TcI and isolated from *Didelphis* sp., were analyzed with 27 microsatellite loci (hence coded by nuclear genes), while a subset of this sample was analyzed with 10 maxicircle loci (that are equivalent to mitochondrial genes) [20]. The double tree obtained (Figure 5) shows that this TcI sample is strongly subdivided into various lesser near-clades, with several significant bootstrap values. Some discrepancies are recorded between the two trees, which can be explained by either occasional introgression [20] or different evolutionary patterns, or both. The main fact is that this TcI sample exhibits a highly detectable phylogenetic signal, with a clear Russian doll pattern.

**Figure 6:**
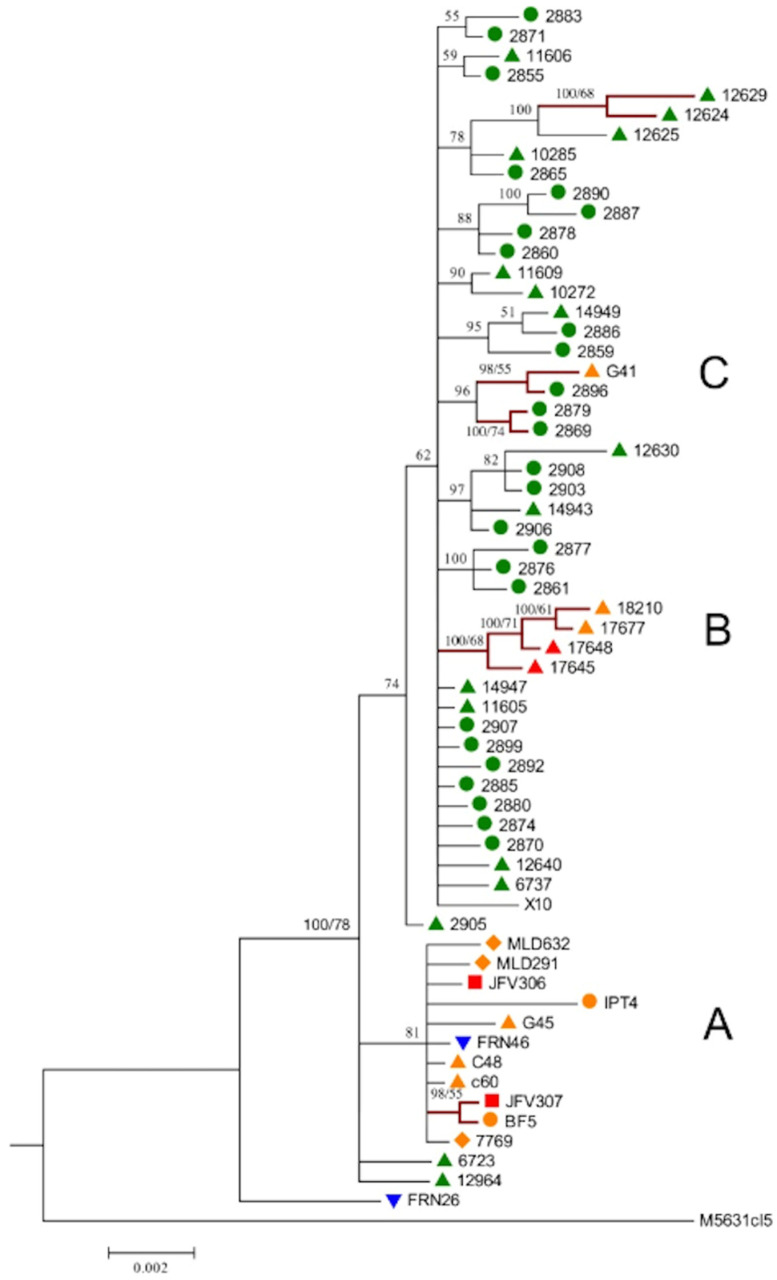
Concatenated Multilocus Sequence Typing (MLST) tree in a sample of TcI Brazilian strains (after [21]). Similarly to Figure 5, a different sampling of Brazilian strains of TcI shows various lesser near-clades within this near-clade. Many of them are supported by bootstrap values that are above the limit of 0.70 used in the present paper [4].

In Brazil, 78 TcI strains isolated from various hosts, including *Didelphis* sp., primates, rodents, bats, triatomine bugs, collected over five ecologically diverse biomes, were analyzed with the sequencing of six housekeeping nuclear genes (Multilocus Sequence Typing or MLST), 25 microsatellite loci and one maxicircle gene (*CO*II), thus combining slow- and fast-evolving markers [21]. The phylogenies based on individual housekeeping genes exhibit moderate levels of incongruence. However, the concatenated tree shows a clear structuration into several lesser near-clades, many of them being supported by significant bootstrap values (Figure 6). This clustering can be explained by neither geographical repartition nor host specificity.

**Figure 7:**
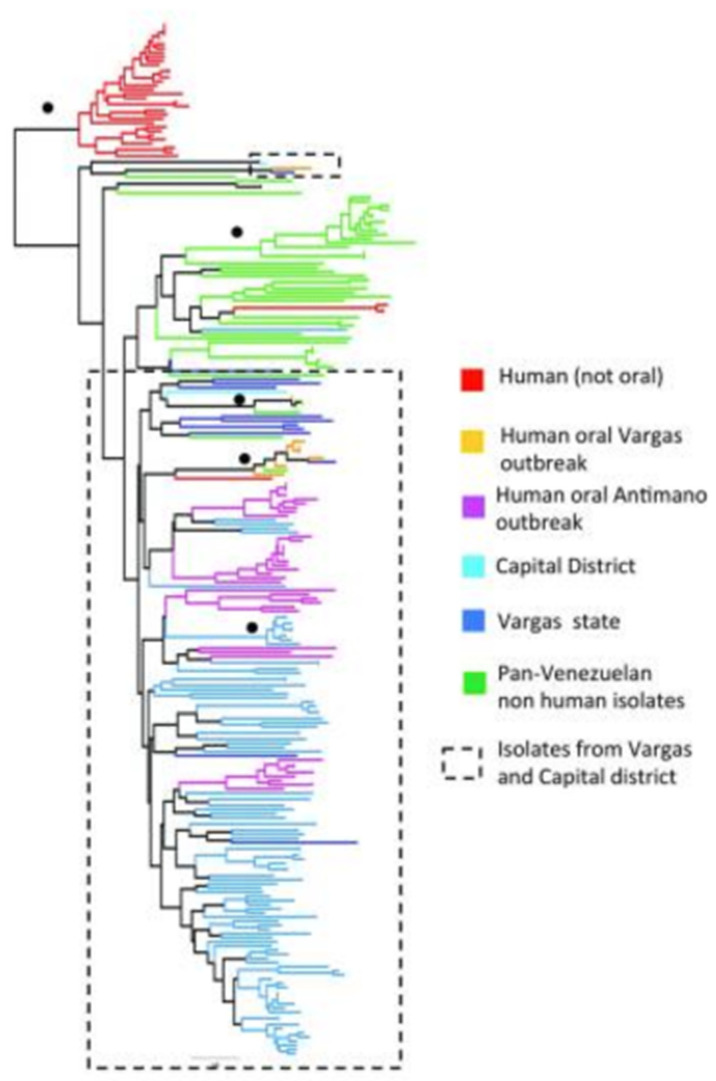
Multilocus microsatellite phylogenetic tree of 246 TcI Venezuelan strains. In Venezuela, the TcI near-clade is also subdivided into many lesser near-clades. Black circles indicate nodes with >60% bootstrap support [22].

In Venezuela, 246 TcI human strains, some of them being isolated after an outbreak of oral transmission, were typed with 23 microsatellite loci [22]. The tree obtained (Figure 7) again shows the presence of various lesser near-clades with several significant bootstrap values.

**Figure 8:**
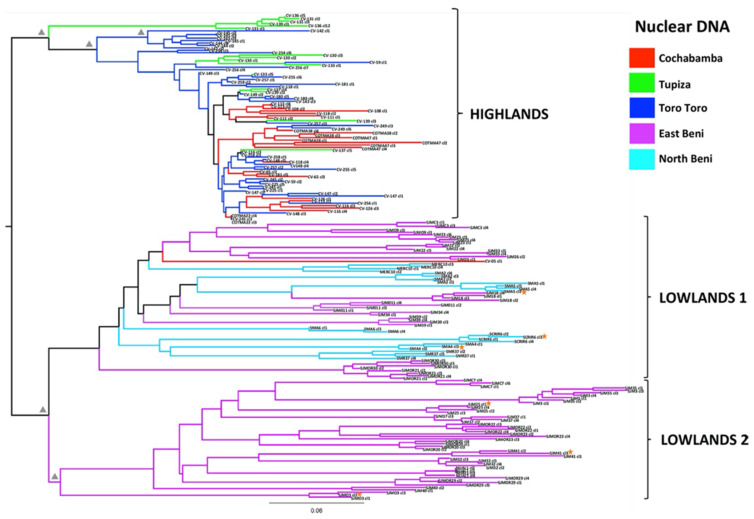
A microsatellite phylogenetic tree of sylvatic TcI strains in Bolivia (after [23]). In Bolivia also, TcI selvatic strains show clustering into many lesser near-clades. Closed grey triangles are adjacent to nodes that receive >60% bootstrap support. Genetic separation accounts only partly for this clustering pattern.

In Bolivia, 199 clones isolated from 68 sylvatic TcI strains from both the lowlands and the highlands of the country were typed with 26 microsatellite loci and 10 maxicircle (=mitochondrial) loci [23]. The microsatellite and maxicircle phylogenies show some discrepancies, which the authors explain by introgression events. However, they broadly agree, which shows that these two very different parts of the genome do not evolve independently (linkage disequilibrium). When microsatellite diversity is considered, high levels of linkage disequilibrium are recorded, including within each subpopulation of the sample. The microsatellite phylogeny shows strong clustering patterns (lesser near-clades) that are not explained by either host specificity or geographical separation (Figure 8).

**Figure 9:**
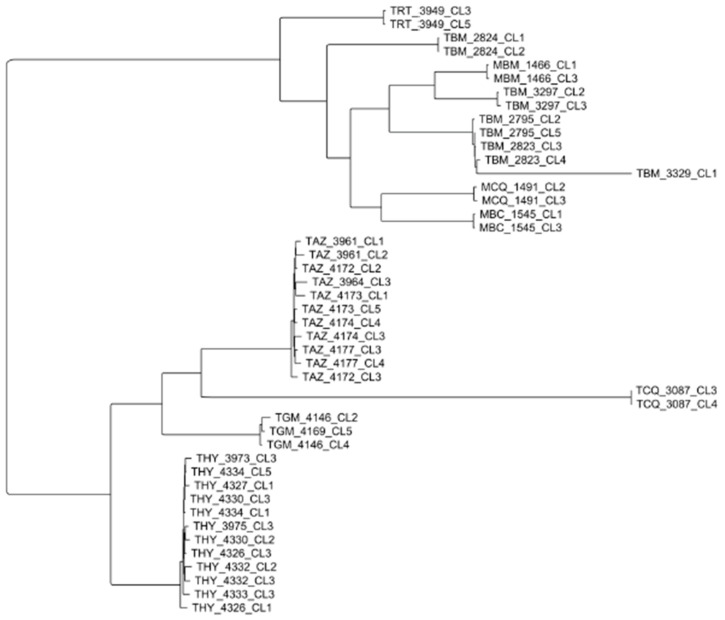
Two lesser near-clades within the TcI near-clade in Ecuador. In spite of clear indications of meiosis in the top cluster, a clear phylogenetic signal is evidenced at the level of the whole sample and within each of the two lesser near-clades (after [6]). “Support is unambiguous for main clusters and high within subclusters, except where last branch lengths are quite short in Cluster 2” (P. Schwabl, personal communication).

In Ecuador, a population genomics survey has revealed within the near clade TcI two distinct genetic clusters (=lesser near-clades) [6]. One shows clear indications of meiosis, whereas the other one does not. However, as already exposed, the isolated observation of meiosis is not in itself sufficient to conclude a panmictic pattern and to challenge the PCE model. As a matter of fact, in [6], (i) the evidence of two distinct clusters (=near-clades) within the near-clade TcI is in itself a Russian doll pattern; (ii) the occurrence of meiosis proves to be an exceptional event (3 meioses/1000 mitoses [6]); (iii) although the difference in population structure between the two clusters is undisputable, the number of different individuals remains weak—eight individuals, since several samples correspond to laboratory clones of the same isolate. This limited sample size leads to the risk of a statistical type II error with possible erroneous hypothesis of panmixia; (iv) in the first population (Bella Maria locality), even if one considers only the eight isolates that are supposed to exhibit meiosis, in spite of this limited sample size, the phylogenetic signal still is highly detectable—“support is unambiguous for main clusters and high within subclusters, except where last branch lengths are quite short in Cluster 2” (P. Schwabl, personal communication) (see Figure 9). This is evidence that genetic exchange is not frequent and not effective enough to erase a clear phylogenetic signal. This is the very definition of PCE. This is even more evident when including the whole Bella Maria population, which comprises an isolate that pertains to the second cluster and is phylogenetically quite distinct—see Figure 9.

When other *T. cruzi* near-clades are considered, within TcII, a phylogenetic signal has been evidenced by genomic data [24]. The study dealt with a limited number (seven) of TCII strains isolated in Minas Gerais (Brazil) and surveyed for both nuclear and mitochondrial genomes. Phylogenies based on the nuclear and mitochondrial genomes show that the majority of branches are shared by both sequences. This gives evidence for the fact that nuclear and mitochondrial genomes do not evolve independently (linkage disequilibrium). The strength of the results is diminished by the limited number of strains. However, clustering (lesser near-clades) is apparent among these strains (Figure 10B in [24]).

**Figure 10:**
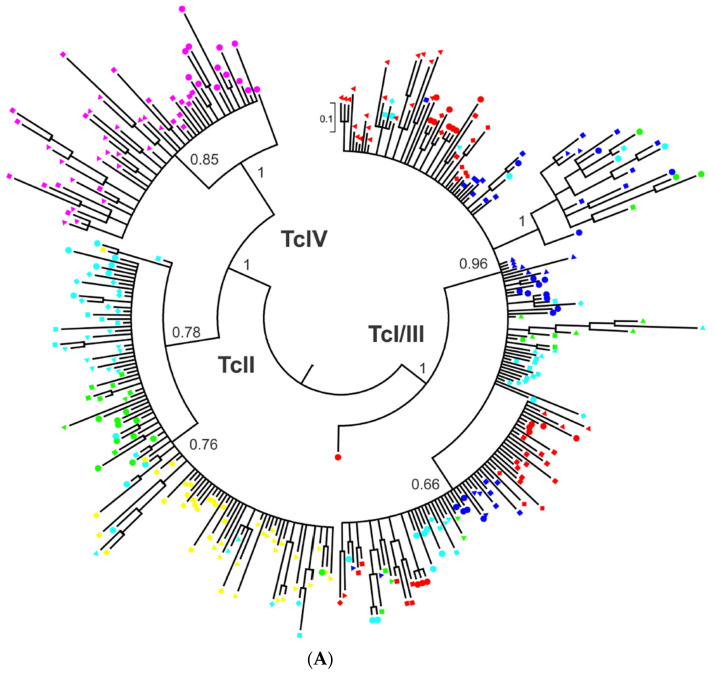

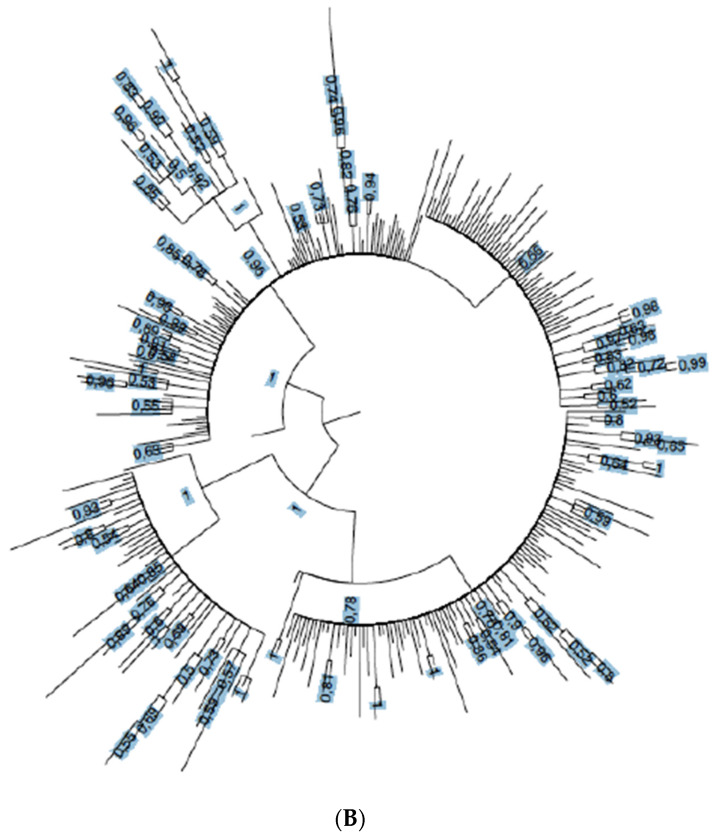
(**A**) The analysis by 335 independent satellite DNA sequences of 19 *T. cruzi* strains reveals various lesser near-clades within each of the six *T. cruzi* near-clades (after [25]). (**B**) (Original figure communicated by J.C. Ramírez). The lesser near-clades within each of the six *T. cruzi* near-clades are supported by highly significant bootstrap values (J.C. Ramírez, personal communication).

Lastly, 19 stocks representative of the 6 *T. cruzi* near-clades (TcI-VI) were analyzed for 335 distinct satellite DNA sequences [25]. The Bayesian phylogeny shows that each of the six near-clades is strongly divided into many lesser near-clades (Figure 10A) with highly significant bootstrap values (Figure 10B).

## 5. Concluding Remarks

Genomics and high-resolution typing data show that evolutionary patterns at a microevolutionary level (within near-clades) look like a miniature picture of the evolutionary pattern of the full *T. cruzi* species. This is especially well ascertained for the near-clade TcI, for which more data are available. However, data from other near-clades are consistent with this Russian doll pattern [15]. The fact that evolutionary patterns are similar at micro- and macroevolutionary scales suggests that the agent of Chagas disease undergoes progressive, gradual, rather than saltatory, evolution.

The indications for meiosis within TcI in Ecuador [6] undoubtedly constitute a very relevant piece of information about *T. cruzi* evolution. However, this does not challenge the hypothesis of a Russian doll pattern within TcI and the PCE hypothesis in *T. cruzi*. As a matter of fact, the existence of occasional bouts of introgression and hybridization at the level of the whole species [23,26,27] does not challenge PCE in *T. cruzi*, since these occasional events do not break the prevalent PCE pattern (presence of a stable and detectable phylogenetic signal and of near-clades). This maintenance of a detectable phylogenetic signal corroborated by various genetic markers (congruence criterion) corresponds to the “clonality threshold”, which is the main trait that specifically gives evidence for PCE [2]. As a matter of fact, beyond this clonality threshold, genetic exchange and recombination are efficiently countered by PCE, and near-clades diverge in an irreversible way. Quite similarly, occasional meiosis events within TcI [6] do not challenge PCE at the within near-clade level, since they do not hamper the persistence of a stable and detectable phylogenetic signal and of lesser near-clades within this near-clade, as clearly evidenced by the many cases exposed in this article (Figure 5, Figure 6, Figure 7, Figure 8, Figure 9 and Figure 10). 

These results show that molecular epidemiology (typing of multilocus genotypes and of lesser near-clades) remains possible within each of the six *T. cruzi* near-clades, since the stability of genotypes is maintained by PCE at this evolutionary level.

It remains to be seen whether genetic clustering and lesser near-clades within each of the six T. *cruzi* near-clades exhibit constant patterns over space and time, in different ecosystems and hosts, and so behave like simili-taxa, a pattern that is observed for example in the yeast *Cryptococcus neoformans* [2].
